# Efficacy of computer-assisted robotic based clinical training program for spinal oncology education on pedicle screw placement

**DOI:** 10.1007/s11701-023-01804-7

**Published:** 2024-04-02

**Authors:** Pengru Wang, Yingye Xin, Shangbin Zhou, Shujie Duan, Danyang Bai, Bo Li, Wei Xu

**Affiliations:** 1 Department of Orthopedic Oncology, Changzheng Hospital, Naval Military Medical University, 415 Fengyang Road, Shanghai, 200003 China; 2 School of Health Science and Engineering, University of Shanghai for Science and Technology, Shanghai, China; 3 Department of Orthopedics, Naval Medical Center, Naval Military Medical University, Shanghai, China

**Keywords:** Pedicle screw placement, Cumulative sum analysis, Learning curve, Spinal oncology education

## Abstract

Pedicle screw placement (PSP) is the fundamental surgical technique that requires high accuracy for novice orthopedists studying spinal oncology education. Therefore, we set forth to establish a computer-assisted robotic navigation training program for novice spinal oncology education. Novice orthopedists were involved in this study to evaluate the feasibility and safety of the computer-assisted robotic navigation (CARN) training program. In this research, trainees were randomly taught by the CARN training program and the traditional training program. We prospectively collected the clinical data of patients with spinal tumors from 1st May 2021 to 1st March 2022. The ability of PSP was evaluated by cumulative sum (CUSUM) analysis, learning curve, and accuracy of pedicle screws. The patients included in both groups had similar baseline characteristics. In the CUSUM analysis of the learning curve for accurate PSP, the turning point in the CARN group was lower than that in the traditional group (70th vs. 92nd pedicle screw). The LC-CUSUM test indicated competency for PSP at the 121st pedicle screw in the CARN group and the 138th pedicle screw in the traditional group. The accuracy of PSP was also significantly higher in the CARN group than in the traditional group (88.17% and 79.55%, *P* = 0.03 < 0.05). Furthermore, no major complications occurred in either group. We first described CARN in spinal oncology education and indicated the CARN training program as a novel, efficient and safe training program for surgeons.

## Introduction

The coronavirus disease 2019 (COVID-19) was declared a pandemic on 11 March 2020, after which millions of lives were lost and healthcare systems around the world were restructured. In real clinical practice, we discovered that acute diseases could be managed by several different medical specialties [1]. Unfortunately, elective surgeries and many other surgical procedures were canceled or postponed in China. The overall orthopedic case volume has also been drastically scaled back. Therefore, as an in-person activity, teaching has been challenging during the COVID-19 pandemic. Although there has been increasing investigation of the orthopedic potential role of multiple education methods, the emergence of the high-risk potential of injury to the spinal cord and vascular structures as well destroying destruction of landmark bony structures due to tumors invading nature, has led to the dilemma in orthopedic oncology teaching. Therefore, because of the challenges associated with the COVID-19 pandemic, alternative surgical methods that allow students the opportunity to gain knowledge and skills are in demand.

With the advent of artificial intelligence and mechanical automation, imaging and computer-assisted robotic navigation (CARN) techniques have been adopted in multidisciplinary surgery. Robotic surgery was first introduced in neurosurgery in 1995, and a robot was first introduced into spinal surgery in the mid-2000s [2, 3]. The robotic system was initially applied in spinal surgery because the freehand technique had a relatively high rate of suboptimal pedicle screw placement (PSP). Several studies [4–6] have already shown the promising outcomes, accuracy, and precision of PSP with computer-assisted robotic systems. Although it has, in theory, shown satisfactory results in training novice surgeons in accurate PSP, this technology is relatively new and has never been used in spinal oncology education.

The objective of this study was to show the application of this technology in spinal oncology education. This new CARN training program for novice orthopedists was developed based on precise screw placement in the real world. To address this, in our present study, we retrospectively explored the learning curve and cumulative summation (CUSUM) for the time to PSP proficiency among trainees.

## Materials and methods

### Participants

**Fig. 1:**
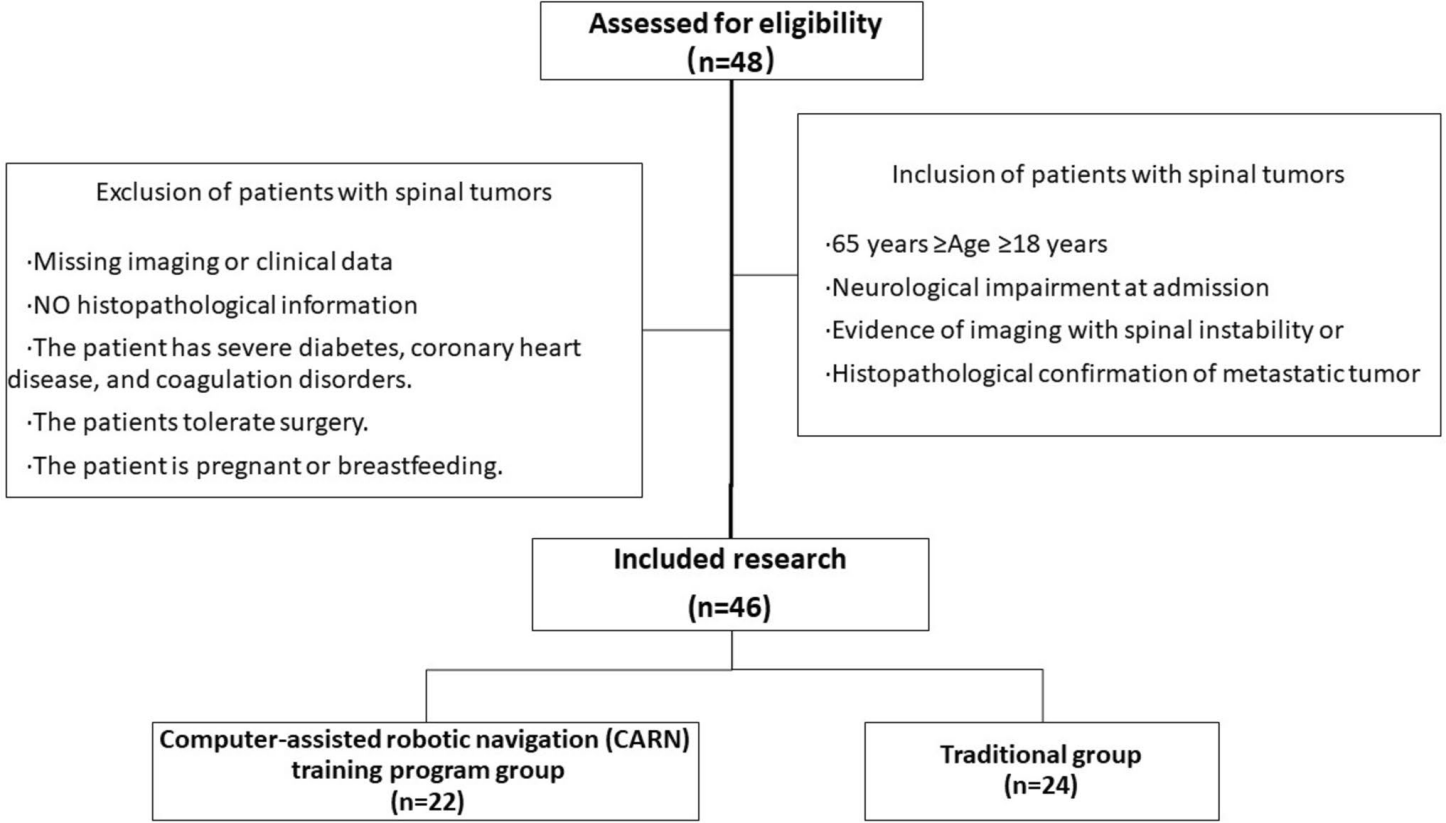
The flow diagram illustrates the process of the computer-assisted robotic navigation (CARN) training program group and the traditional group. The pedicle screws placed by CARN in 22 patients and placed by traditional method in 24 patients were included for learning curve analysis in our study

Two trainees who received the same prior training and had basic knowledge of robotic systems, such as cadaveric courses and instructional didactics, participated in the CARN training program in the Department of Orthopedic Oncology of our hospital. Then, two trainees were randomly taught by CARN and the traditional training program. During this period, 48 patients diagnosed with spinal tumors were included and divided into two groups, while three patients were excluded owing to their postoperative or general situations (Fig. 1). All patients were informed of the research purposes and the potential risks and benefits of the procedure, and provided informed written consent for participation in the procedures as well as their data to be used for further research purposes. All procedures followed were in accordance with the ethical standards of the responsible committee on human experimentation and the Declaration of Helsinki [7]. The study also received local ethics board approval at our hospital.

All clinical data were prospectively recorded from 1st May 2021 to 1st March 2022, including the time to PSP proficiency, serving as primary outcomes, intraoperative failure, and information on intraoperative adverse events. The time to PSP proficiency was recorded prospectively by a robotic-assisted system and note keepers. Intraoperative failure was judged by fluoroscopy examination. Information on intraoperative adverse events was recorded by the engineer and surgeons according to the adverse event standard [8].

### Computer-assisted robotic navigation (CARN) training program

**Fig. 2:**
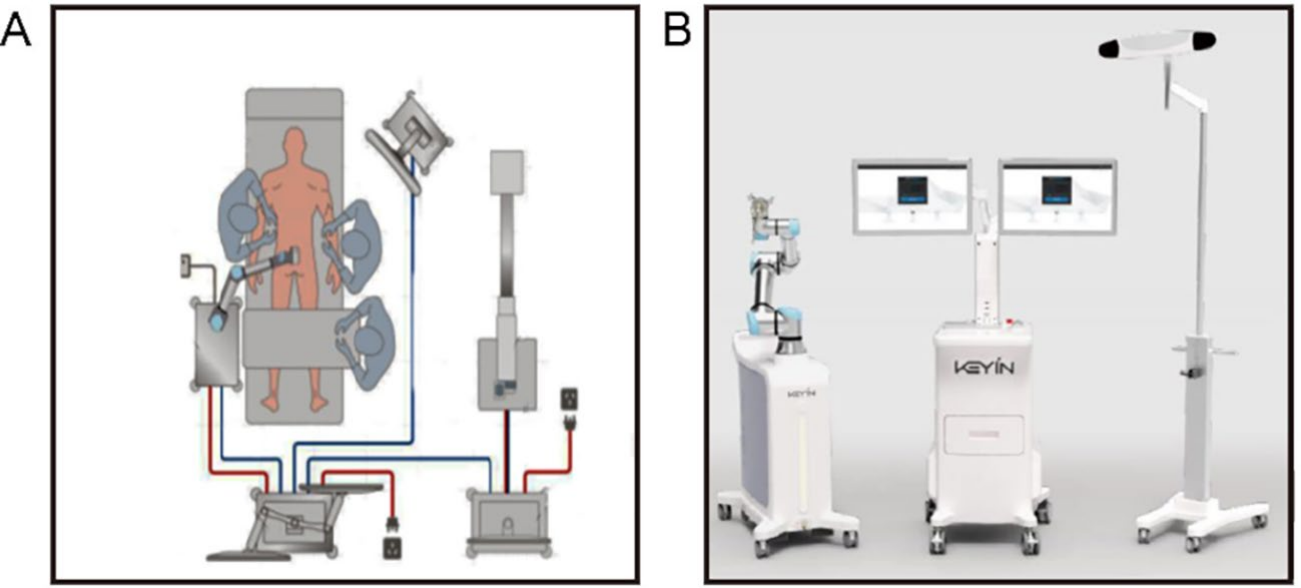
**A** Schematic diagram of CARN in operation. **B** The CARN is mainly composed of a surgical robotic arm, an optical tracking device, and controlling workstation

The CARN training program is composed of a mechanical device and full navigation capabilities. It allows precise PSP by preoperative and intraoperative planning, spatial registration, and sensing technology. All the system components are shown in Fig. 2. Preoperative planning for PSP was decided by professors and trainees using proprietary software for preoperative CT scans. During the operation, the mounting anchor platform was first fixed on the spinous process or ilium. Then, anteroposterior and oblique radiographs were obtained and matched with the preoperation images of the target segment. After rechecking the preoperative plan established by the software, the robot was then linked to the platform and used to direct the instruments to place the pedicle screws according to the preplanned trajectories instead of the anatomical landmark. After the bone surface was exposed, the tool guide and sleeve were brought to the destination. Then, the trainees placed and corrected the position of the pedicle screws with a positioning tool under navigation guidance. If the pedicle screws were placed improperly, the feedback system would signal the prewarning system. The positioning tool would brake immediately and correct the position of the pedicle screw to the planned site. In the traditional cohort, pedicle screws were placed by the normal apprenticeship model, hand by hand.

### Evaluation of teaching methods—cumulative sum analysis of the learning curve and evaluation scale for the CARN training program

The learning curve is a term that describes how a skill would be acquired by a particular practice for an extended period [9]. Previously, Urakov et al. [10] described that as caseload increased, the proficiency of the neurosurgeon dramatically improved in the early stage and then plateaued. Since being mentioned in the 1970s, it has been proven to be a substantial guideline for surgical education and cost‒benefit decisions for novel surgical methods. However, these learning curves could not show the turning point in the education process, so the CUSUM test was designed for monitoring quality control [11]. The CUSUM test was also regarded as a stability test of the target level of technical competence. Therefore, the CARN training program is well suited for ongoing feedback on surgical training and for the CUSUM test to invigilate processes [12, 13].

Therefore, in our research, the CUSUM analysis was first adopted for the learning curve for the time to PSP proficiency in robotic surgery. The CUSUM score is shown in the line graph, with the *X*-axis showing the procedures and the *Y*-axis representing the CUSUM scores:

$$\begin{gathered} {\text{CUSUM}}\;C_{n} = C_{n - 1} + \left( {C_{n} - \mu } \right)\quad (n = 1,2,3, \ldots ,n) \hfill \\ {\text{RA-CUSUM}}\;Y_{n} = Y_{n - 1} + (T_{n} - S)\quad \left( {n = 1,2,3 \ldots ,n} \right). \hfill \\ \end{gathered}$$

According to CUSUM analysis, four parameters should be defined as the acceptable failure rate (*p*_0_), the unacceptable failure rate (*p*_1_), the type I error rate (*α*), and the type II error rate (*β*). The *α* and *β* rates were set at 0.05 and 0.20, respectively, in the two CUSUM analyses. *μ* represents the mean value of the PSP time. The cases in the research were distributed chronologically, with the first case being assigned the number 1, with each subsequent case being assigned a successive number. In this formula, “*T*_*n*_ = 0” represents success, while “*T*_*n*_ = 1” refers to the failure of performance. The standard line for the pedicle screw was set as 6.5 min, serving as the time for PSP at a single segment by trainees. When the time was less than or equal to 6.5 min, the performance was considered successful, while a time greater than 6.5 min was defined as procedural failure. Failure also refers to the situation where trainees could not place the pedicle screws or when the inner walls of the pedicle were coloboma. In this study, the presignified standard of performance was derived from previous operation experience. For the time of the pedicle screw, the acceptable and unacceptable failure rates were scored as 20% and 40%, respectively. Table 1 summarizes the details used in the CUSUM charting protocol for monitoring pedicle screw fixation.

**Table 1:** Formulas and values involved in plotting the learning curve cumulative summation test

Variable	Value
*p*_0_, unacceptable failure rate	0.4
*p*_1_, acceptable failure rate	0.2
*α* probability of the type I error	0.05
*β*, probability of the type II error	0.2
*P* = ln(*p*_1_/*p*_0_)	− 0.6932
*Q* = ln[(1 − *p*_0_)/(1 − *p*_1_)]	− 0.2877
*S* = *Q*/(*P* + *Q*)	0.2933
1 − *S*	0.7067
*a* = ln[(1 − *β*)/*α*]	2.77
*H* = *a*/(*PþQ*), decision limit	− 2.83

### Data analysis

The normality distribution of the data was analyzed using a Shapiro‒Wilk test. Continuous variables are expressed as the mean (SD), and categorical variables are expressed as frequencies. The Wilcoxon rank sum test and Chi-squared test were used to compare differences between the groups. The functional relationship between the time to PSP proficiency and the case number was fitted using a smoothing plot. Significance was set at *P* < 0.05. Statistical analysis was conducted using SPSS 26 and GraphPad Prism.

## Results

### Patients

In our study, we enrolled 45 consecutive patients who were clearly diagnosed with spinal cancers and needed surgical treatments between 1st May 2021 and 1st March 2022. Eleven (50%) were men and 11 (50%) were women in the CARN group and 11 were men (47.83%) and 12 were women (52.17%) in the traditional group. The mean age was 61.14 ± 10.00 and 55.52 ± 13.52 in the CARN and traditional groups, respectively (*P* = 0.88). Participants in both groups had similar baseline characteristics, as shown in Table 2.

**Table 2:** Descriptive statistics of the subjects in the study

Characteristic	CARN group^a^	Traditional group	*P* value
Age, mean ± SD, (year)	61.14 ± 10.00	55.52 ± 13.52	0.18
Females, *n* (%)	11 (50%)	12 (52.17%)	0.88
BMI, mean ± SD, (kg/cm^2^)	19.56 ± 2.07	22.11 ± 2.76	0.22
Diagnosis (*n*)	22	23	
Lung	13	12	0.64
Liver	2	3	0.67
Multiple myeloma	3	4	0.73
Prostate cancer	2	1	0.52
Renal carcinoma	1	1	0.97
Parotid carcinoma	1	1	0.97
Rectal cancer	0	1	–
Operative level (*n*)			
Thoracic vertebra	14	14	0.85
Lumbar	8	9	

^a^*CARN* computer-assisted robotic navigation training program

### The learning curve and the efficacy of the CARN training program

**Fig. 3:**
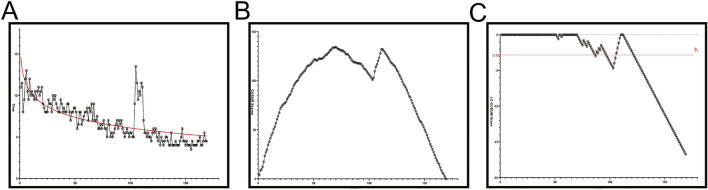
**A** Scatterplot of the operation time in CARN group according to surgeon experience. The smooth curve line represents logarithmic approximation curves. **B** CUSUM score analysis for operating time in the 22 patients who underwent CARN for 169th pedicle screw. The 70th pedicle screw shows the occurrence of a turning point in the learning curve. (*CUSUM* cumulative sum). **C** Cumulative summation test for learning curve signaled competency after 121st screws. When the time was less than or equal to 6.5 min, the performance was considered as success

**Fig. 4:**
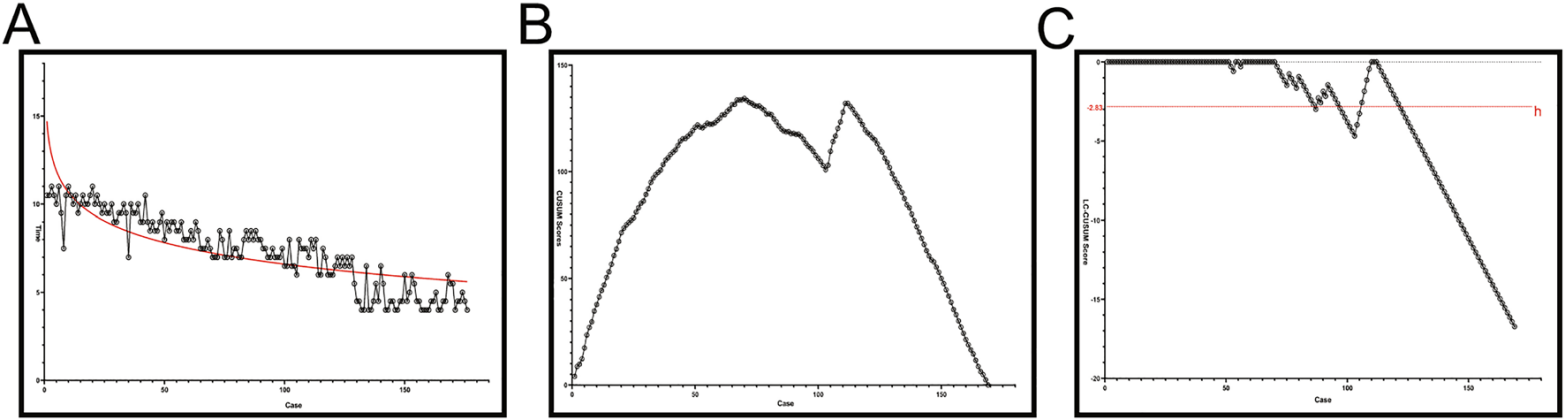
**A** Scatterplot of the operation time in traditional group according to surgeon experience. The smooth curve line represents logarithmic approximation curves. **B** CUSUM score analysis for operating time in the 24 patients who underwent traditional method for 175th pedicle screw. The 92nd pedicle screw shows the occurrence of a turning point in the learning curve. (*CUSUM* cumulative sum). **C** Cumulative summation test for learning curve signaled competency after 138th screws. When the time was less than or equal to 6.5 min, the performance was considered as success

The fitted formula of a trainee’s learning curve educated by the CARN training program is *Y* = 14.61–4.267 × log(*x*). The goodness of fit is *R*^2^ = 0.5713 (Fig. 3A). As shown in the CUSUM graph (Fig. 3B), the first 70 pedicle screws were placed in the early stage, which represents the learning period of the new technology. From the 71st to 169th cases, the curve was flat and continued to decline, representing the mastery of the surgical technique. The mean time to PSP proficiency in the late stage (6.58 ± 1.84 min) was shorter than that in the early stage (9.84 ± 1.41 min). The LC-CUSUM test signaled competency for PSP at the 121st pedicle screw. Before the final one, there are also ten times reaching the satisfactory level (Fig. 3C). The other trainee participated in the traditional education model in surgery practice. He completed 175 pedicle screws in the spine. The fitting formula was *Y* = 14.72–4.056 × log(*x*). The goodness of fit is *R*^2^ = 0.6685 (Fig. 4A). According to the shape of the learning curve, the learning curve of normal could also be divided into two stages: the first 92 cases were in the early stage, and the 93rd to 175th cases were in the late stage (Fig. 4B). The operation time during the late stage (6.47 ± 1.51 min) was shorter than that during the early stage (9.90 ± 1.16 min). Figure 4C shows the ability of PSP. The LC-CUSUM test signaled competency for PSP at the 138th pedicle screw, and there was also no time of satisfactory results before the 138th pedicle screw.

### Accuracy of the pedicle screw and the safety of the CARN training program

A total of 167 and 176 pedicle screws placed into the thoracolumbar spine were evaluated for accuracy by CT scans. Overall, in the CARN training program group, 149 (88.17%) pedicle screws were evaluated as Grade A according to the Gertzbein–Robbins classification [14]. There were 20 pedicle screws (11.83%) classified as grade B. In the traditional group, a perfect trajectory was observed in 140 (79.55%) PSPs. The rate of perfect trajectory was significantly higher in the CARN training program group than in the traditional group (*P* = 0.03). For the remaining screws, 20.45% (*n* = 36) were grade B. Grade A or grade B pedicle screws were regarded as “clinically acceptable screws”. None of the pedicle screws was shown as C or D grades in either group. The acceptable rates of the CARN group and the traditional group both reached the perfect rates (Table 3).

**Table 3:** The grade of pedicle screw placement (PSP) in patients with spinal tumor in two groups

Grade of PSP	CARN group	Traditional group	*P* value
A *n* (%)	149 (88.17%)	140 (79.55%)	0.03
B *n* (%)	20 (11.83%)	36 (20.45%)	
C *n* (%)	0	0	
D *n* (%)	0	0	

In the early stage of the CARN training program group, 58 (82.86%) of the first 70 pedicle screws were graded as the A level. Seventy-two (78.26%) of the 92 pedicle screws taught by the traditional method were evaluated as Grade A. In the late stage, the rate of grade A was 89.88% (71 grade screws) and 80.95% (84 grade screws) in both groups. These perfect trajectory pedicle screws tended to show more in the CARN group, but they did not reach statistical significance. Furthermore, no major complications, such as superficial or deep infection, neurovascular injury, or life-threatening complications, occurred.

## Discussion

There used to be several complex barriers facing an intern to become a real orthopedist. In addition, the advent of the COVID-19 pandemic has led to a sharp reduction in surgical ability, and opportunities to practice orthopedic surgeries. It has now become more difficult for interns to learn procedures in clinical practice than previously during residency training. Admittedly, adapting to the “post-COVID-19 Era” is still a stressful and ongoing challenge. Therefore, the approach to teaching should also be changed in terms of methods, especially for surgical interns.

As a spinal surgeon, PSP is a fundamental surgical technology that provides multidimensional protection and significant rigidity for further treatment [15]. Traditionally, pedicle screws in spinal oncology patients have been inserted by hand, but pedicle screws inserted by hand are often inaccurately placed due to the complex anatomical structure of the spinal tumor based on the anatomical site. Therefore, inaccurate placement of pedicle screws may damage vital tissues, leading to serious surgical complications, including pedicle fracture or penetration, nerve root or spinal cord injury, vascular injury, dural rupture, and epidural hematoma. In patients with spinal tumors, radiation and chemotherapy also severely impair osseous healing and reduce bone quality. Therefore, once screws are malposition in frail patients, the risks of spinal fracture and instability are even higher. The desire to decrease the time to proficiency and the incidence of surgical complications while increasing efficiency and accelerating training seem to be more critical and urgent than at any time before the COVID-19 pandemic.

In a previous study of spinal surgery, the safety of PSP by primary surgeons using the freehand technique was acceptable under appropriate supervision [16]. Some studies also reported that as PSP training began, accuracy and operation time finally tended to reach a plateau [17]. However, because of differences in medical education and surgical practice, the turning point was also different in different studies. Gonzalvo et al. [18] showed that a significant decline in complication rate and a sharp reduction in the operation time of PSP occurred after approximately 80 screws were placed, approximately in the 25th patient. In another study, according to the LC-CUSUM analysis, the turning point on the learning curve marking adequate placement was approximately 114 screws in the 17th patient. K. J. Ryu [19] also described a continuous decreasing trend in accurate screw placement until 23–25 patients were operated on. Other papers [20–22] also showed a similar learning curve for spinal surgeries to correct a deformed spine, but they did not report a definitive number of PSPs needed to achieve proficiency. They also focused more on scoliosis surgery but seldom on tumor surgery.

Computer-assisted navigation techniques have shown promising results by increasing the accuracy of spinal instrumentation, reducing the risk of potential complications, and reducing radiation exposure [23, 24]. It is also possible to achieve a high degree of precision with the technology and repetitive tasks can be completed without diminishing performance. However, although there are limitations in visibility and surgeons’ experience after participating in a traditional training program, computer-assisted robotic systems may be an ideal teacher for PSP. Our most important finding was the steeper learning curve seen in studies examining surgical training methods associated with the robotic program. We found that the CARN training program helped novice orthopedists become proficient in PSP with fewer pedicle screws and fewer patients. Furthermore, the learning curve associated with the CARN training program was steeper than both the traditional PSP training program and the post-report [25] according to our findings. In addition, the decline in the time to PSP proficiency in the early stage was more visible in CARN education than in traditional education even if the difference did not reach statistical significance. Compared with the traditional education group, the participants in the CARN group were able to reduce the time to proficiency for spinal surgery. However, the mean time to proficiency was longer in the CARN group. It seems that spine surgeons with experience in PSP would not benefit so much from the CARN training program.

As expected, the CUSUM test was successful in evaluating the quality of our new educational model. In our research, these plots did cross the satisfactory level of h. The results may indicate that the CARN training program and the traditional freehand training program would lead to accurate PSP. Even if CARN was a new training system for surgeons, implementation of this novel training program showed a favorable result considering that all the trainees were less experienced spine surgeons. In the early stage of LC-CUSUM, pedicle screw failure was observed more often in our study in both trainees. Therefore, learning curves would be flattened as they started from a well-educated point in real surgical practice than students did in our study. The safety of the CARN training program was also evaluated by the accuracy of pedicle screws. The rate of perfect trajectory, Grade A, was statistically higher in the CARN training program group than in the traditional group. In our research, no adverse events or revision surgeries were recorded.

We acknowledge the following limitations of our study. First, it was based on only two trainees’ surgical practices at a single institution. In addition, the two spinal surgeons were familiar with this new robotic-assisted training program. Therefore, this learning curve and CUSUM curve may be more appropriate for those well-versed in courses focusing on computer-assisted robotic systems. Second, all the procedures performed in this study were open surgeries with full exposure of the relevant anatomy. Therefore, we were unable to evaluate the accuracy of the CARN training program when used for percutaneous or minimally invasive spine procedures.

## Conclusion

The learning curve of CARN showed that approximately 70 pedicle screws were needed to achieve mastery of PSP compared with 92 pedicle screws in the traditional training program. Through this experiment, we not only confirmed the advantage of the CARN training program for PSP but also indicated that is an efficient and safe surgical training program for spine surgery even when a crisis affects the health care organization.

## Data Availability

Not applicable. The datasets used and/or analyzed during the current study are available from the corresponding author on reasonable request.
